# Migratory Whooper Swans *Cygnus cygnus* Transmit H5N1 Virus between China and Mongolia: Combination Evidence from Satellite Tracking and Phylogenetics Analysis

**DOI:** 10.1038/s41598-018-25291-1

**Published:** 2018-05-04

**Authors:** Shuhong Li, Weiyue Meng, Dongping Liu, Qiqi Yang, Lixia Chen, Qiang Dai, Tian Ma, Ruyi Gao, Wendong Ru, Yunfeng Li, Pengbo Yu, Jun Lu, Guogang Zhang, Huaiyu Tian, Hongliang Chai, Yanbing Li

**Affiliations:** 1 Key Laboratory of Forest Protection of State Forestry Administration, National Bird Banding Center of China, Research Institute of Forest Ecology, Environment and Protection, Chinese Academy of Forestry, Beijing, 100091 China; 2 State Key Laboratory of Remote Sensing Science, College of Global Change and Earth System Science, Beijing Normal University, Beijing, 100875 China; 3 Chengdu Institute of Biology, Chinese Academy of Sciences, Chengdu, 610041 China; 4 National Urban Wetland Park of Sanmenxia Swan Lake, Sanmenxia, 472000 China; 5 Shaanxi Provincial Centre for Disease Control and Prevention, Xi’an, 710054 Shaanxi China; 6 College of Wildlife Resources, Northeast Forestry University, Harbin, 150040 China; 7 Harbin Veterinary Research Institute, Chinese Academy of Agricultural Sciences, Harbin, 150040 China

## Abstract

In late 2014, a highly pathogenic avian influenza (hereafter HPAI) H5N1 outbreak infected whooper swans *Cygnus cygnus* wintering at the Sanmenxia Reservoir area, China, and raised concerns about migratory linkages between wintering and breeding grounds of whooper swans. In this study, 61 swans were satellite tracked from 2013 to 2016 to determine the spatial association of their migration routes and H5N1 outbreaks, and 3596 fecal samples were collected along the migration routes for virology testing. Swans departed the wintering grounds and migrated along the Yellow River, and flew over the Yin Mountains in China. The Brownian bridge movement model showed there was a high degree of spatiotemporal overlap between the core use area along the spring migration pathway and historical H5N1 events in China and Mongolia from 2005 to 2015. The H5N1 strain was isolated and phylogenetic analyses confirmed that the HA gene sequence generated is genetically similar to that of the epidemic strain at a previous wintering site (the Sanmenxia Reservoir area) along its flyway. Our results identified a previously unknown migratory link of whooper swans in central China with Mongolia and confirmed that the swans could carry the HPAI H5N1 virus during migration, resulting in long-distance transmission.

## Introduction

Wild birds are a natural reservoir for avian influenza virus^1^. The highly pathogenic avian influenza (hereafter HPAI) H5N1 virus of wild birds in East Asia has continued to circulate worldwide since 2005, when it first gained considerable attention after causing the mortality of more than 6000 wild birds at Qinghai Lake, China^2,3^. Migratory waterfowl, in particular the Anatidae family, have been suspected as the primary species that carries various subtypes of H5N1 between their wintering and breeding grounds every year^4–6^. Their long-distance travel contributes to the transmission of H5N1 into the Far East of Russia^7^ and even into Europe and Africa^8,9^. It is widely hypothesized that migratory birds play a global role in the dispersal of H5N1^10–13^.

Whooper swans *Cygnus cygnus* in eastern Asia winter in China^14^, South Korea^15^ and Japan^16^. Their breeding range extends from the Gobi Desert wetlands of westernmost Mongolia to the tundra rivers of easternmost Russia, as evidenced from banding and satellite data^17^. In recent years, whooper swans have drawn attention as sentinel species in relation to H5N1 dissemination^15–17^. H5N1 clades 2.2 and 2.3.2 were detected successively in dead whooper swans from 2005 to 2010 in Mongolia^17,18^. Additionally, clades 2.3.2 and 2.3.2.1 of the H5N1 subtype was found in whooper swans in 2008 and 2011, respectively, in Japan^19,20^. In early 2015, 93 whooper swans, wintering in the Sanmenxia Reservoir area of Henan, China, were infected with H5N1 clade 2.3.2.1c^21^, suggesting that whooper swans as susceptible wild birds to viruses may play a key role in the geographic spread of H5N1.

The Sanmenxia Reservoir area, in central China, is located at the intersection of the Central Asian Flyway and the East Asian Flyway. Thousands of whooper swans winter in the reservoir area every year. However, information on the detailed migration of whooper swans wintering in central China, H5N1 surveillance along their migration route, and the spatiotemporal association of whooper swan migration and H5N1 outbreaks are still poorly understood, and they are factors that are crucial to understand in terms of the potential transmission process of H5N1.

In this study, we monitored the wintering population dynamics of whooper swans at the Sanmenxia Reservoir area from 2010 to 2016 to evaluate the impact of the H5N1 outbreak that was in the early winter of 2015. We satellite tracked 61 whooper swans wintering at Sanmenxia during 2013 to 2016 to determine their spring migration routes, key stopovers and breeding sites in East Asia. Based on historical H5N1 outbreak data, we analyzed the spatiotemporal relationship of whooper swan migration paths with H5N1 outbreaks. We also collected fecal samples along the migration routes for virology testing. To trace the genetic lineage of the isolates, nucleotide sequences were determined and phylogenetically analyzed. Thus, we provided information on the role of migratory whooper swans in the transmission of the H5N1virus based on a combination method of satellite tracking and phylogenetics analysis.

## Results

### Wintering population dynamics

**Figure 1:**
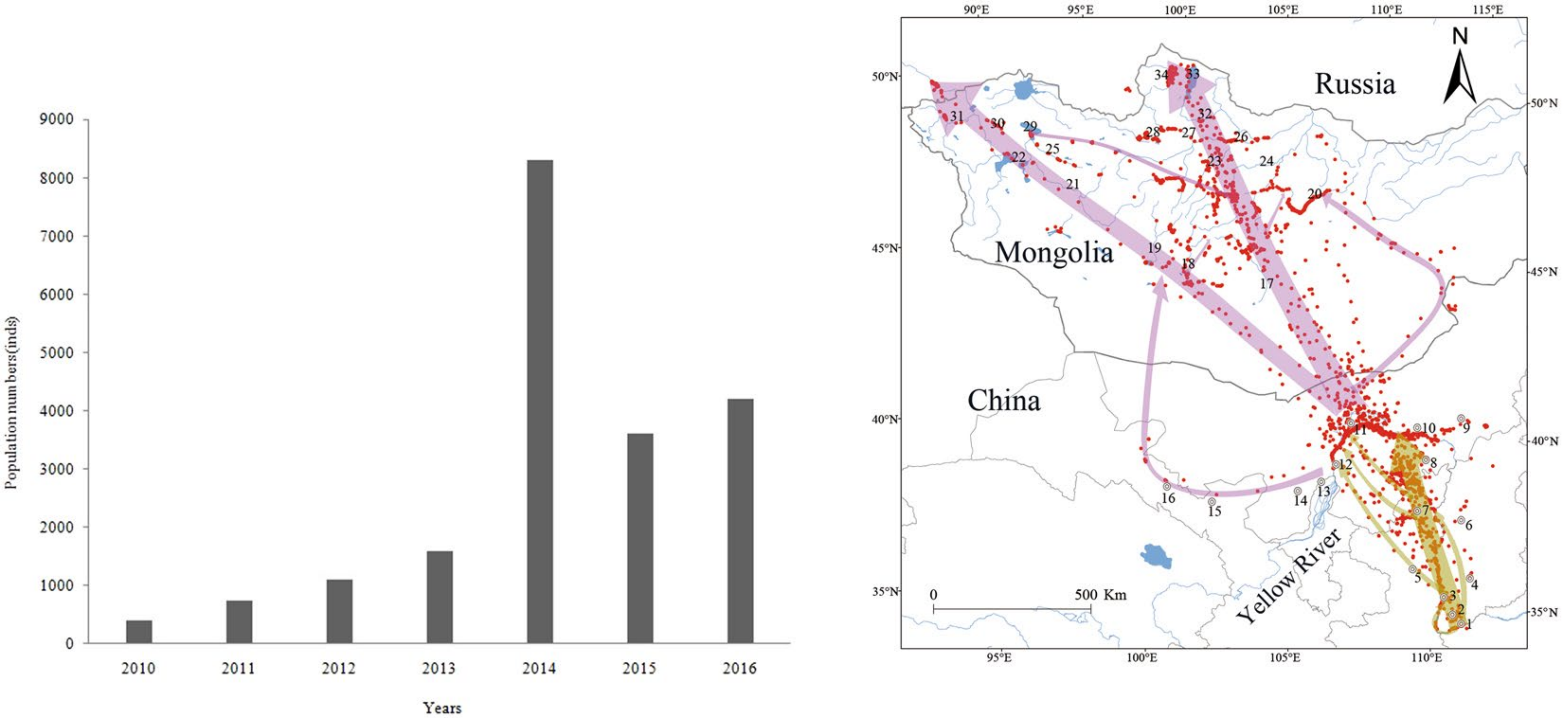
Spring migration routes and population number dynamics of whooper swans wintering at the Sanmenxia Reservoir area in China from 2010 to 2016. 1. Sanmenxia Reservoir area (wintering grounds); 2. Yuncheng; 3. Yumenkou; 4. Linfen; 5. Yanan; 6. Lishi; 7. Yulin; 8. Ordos; 9. Huhehaote; 10. Baotou; 11. Bayan Nur; 12. Wuhai; 13. Shizuishan; 14. Alxa Left Banner; 15. Jincang; 16. Zhangye;17. Ongiyn Gol; 18. Ulungur Lake; 19. Tuin River and Buuntsagaan Lake; 20. Tuul Gol; 21. Dzavhan Gol; 22. Khara Us Nur and Khara Nor; 23. Hanuy Gol; 24. Orkhon River; 25. Hunguy Gol; 26. Selenga River; 27. Delger-Muron River; 28. Sangiin Dalai Lake; 29. Khyargas Lake; 30.Uvsiin Khar Us Lake; 31. Khovd River; 32. Egiyn Gol; 33. Khovsgol Lake; and 34. Shinshkhed Gol. Brown and purple lines indicate the first and second stages, respectively.

The most stable period for the wintering population of whooper swans in the Sanmenxia Reservoir area was from late December to early January. From 2010 to 2016, there were no disease outbreaks, except for the H5N1 outbreak in 2014, and no whooper swans died. The population of whooper swans wintering at the Sanmenxia Reservoir area increased rapidly from 410 (1.58 ind./ha) in 2010 to 8317 (32.11 ind./ha) by 2014. However, after the H5N1 outbreak resulting in the death of 93 swans in winter of 2014, the population size was reduced to 3605 and 3588 in 2015 and 2016, respectively (Fig. 1).

### Migration routes

All tracked swans successfully departed from the wintering grounds during the spring from 2014 to 2016. Of the 61 tracked swans, 31 reached breeding sites in central-northern and western Mongolia, ten spent the summer in China (six in the Yellow River stretch in Inner Mongolia, two in the Ordos Lakes and one in Hongjian Nur), and three were confirmed dead for unknown reasons, with their transmitters found in the Yellow River (S24), Hekou Reservoir (S44) and Hongjian Nur (S47). Transmission from the remainder (n = 27) ended after the birds reached the China–Mongolia border. The total average distance from wintering to breeding grounds was 2081.13 ± 372.25 km (n = 31).

Departure dates from the wintering grounds ranged from 17 February to 27 March (median 7–8 March, n = 63). Arrival dates at the breeding sites in Mongolia ranged from 27 February to 23 May (median 9–10 April, n = 22).

Whooper swans migrated along the Yellow River of Shanxi, Shaanxi and Inner Mongolia and its tributaries (including the Yan River, Hekou Reservoir, Tuwei River), flew over the Yin Mountains in China, and then flew to the rivers and lakes of central-northern and western Mongolia. We summarized the spring migration routes into two stages as follows:

#### From the wintering grounds to the Yellow River stretch in Inner Mongolia in China

The majority of the whooper swans (n = 57) departed from the Sanmenxia Reservoir area and flew northwest directly to the Yumen Estuary. With a short stopover (mean 3.0 d, n = 31) period, they migrated north along the Yellow River, then they took a longer stop at the Yulin wetlands (mean 4.3 d, n = 15) and Ordos wetlands (mean 8.5 d, n = 12) before arriving at the Yellow River stretch in Inner Mongolia for their longest stay (mean 24.0d, n = 43)

Some whooper swans took tributary migration routes. Eight swans departed from the Sanmenxia Reservoir area and migrated west along the upstream of the Yellow River; two swans traveled directly north by way of Linfen and Lishi; fifteen swans departed from the Yumen Estuary (n = 6) and the Yulin(n = 4) and Ordos (n = 5) wetlands following a northwest route that bypassed the Maowusu Sandy Land and Kubuqi Desert, and then they finally arrived at the Yellow River stretch in Inner Mongolia.

#### Departing from the Yellow River stretch in Inner Mongolia to the breeding sites in Mongolia

The majority of the whooper swans (n = 31) departed north directly from the Yellow River stretch in Inner Mongolia in China to Mongolia. One whooper swan migrated west by way of Shizuishan, Alxa Left Banner, Jincang and Zhangye. Migration trends in Mongolia mainly covered three routes.

**Route 1:** Approximately half of the whooper swans (n = 16) flew directly north to the river basins in central-northern Mongolia. Of these swans, two remained at Ongiyn Gol for the entire summer, eight stayed near the Orkhon River, Hanuy Gol and Selenga River, and three moved to the northernmost lakes of the Shinshkhed River basin (including Khovsgol Lake and Dood Tsagaan Lake). Only one swan migrated west along the northern Khangai Mountains into Khyargas Lake in western Mongolia after a short stay at the Orkhon River basin.

**Route 2:** Eleven swans used a northwestern route along the valley between the Khangai Mountains and Govi-Altayn Nuruu. Of these 11 swans, four spent the summer in the Baydrag Gol (including Buuntsagaan Lake) and the Tuin River (including Ulungur Lake). Two stayed at Buuntsagaan Lake and Ulungur Lake for short periods (5 d and 7 d, respectively), and then, they tracked northeast across the Khangai Mountains and into the Orkhon River basin. Five swans continued to migrate along the Zavkhan River before they arrived at the Khovd River basin (Harus Nur, Uvsiin Khar Us Lake, Khara Us Nur and Khara nor) in western Mongolia, even migrating to the boundary between Russia and Mongolia. (Fig. 1).

**Route 3:** A small proportion of swans (n = 3) traveled northeast and retraced their paths to Tuul Gol for the summer.

### Key stopovers and breeding sites

As shown in Table 1, most whooper swans stayed at the Yellow River stretch in Inner Mongolia from 20 February to 6 May (n = 61), the Yumen Estuary from 27 February to 28 March (n = 36), the Ordos Lakes from 25 February to 2 May (n = 23), the Hekou Reservoir from 25 February to 7 April (n = 16), and Hongjian Nur from 19 February to 15 April (n = 14). More than 10 whooper swans stopped at these sites and stayed for 3 days or longer. Thus, we identified these sites as the key stopover sites during the spring migration of whooper swans in China.

**Table 1:** Stopover and breeding sites of the spring migration routes of whooper swans in China and Mongolia. S: Stopover site. B: Breeding site.

Key sites	Stopover/breeding sites	No. of swans	Duration	Arrival date (median date)	Departure date (median date)
Yellow River stretch Inner Mongolia	S	58	26	2/20-4/26,3/12	2/25-5/6,4/3
Yumen Estuary	S	36	5	2/17-3/28,3/9	2/23-3/28,3/12
Ordos Lakes	S	23	22	2/25-4/11,3/14	2/25-5/2,3/23
Hekou Reservoir	S	16	16	2/25-5/7,3/7	2/27-4//7,3/12
Hongjian Nur	S	14	32	2/19-4/13,3/8	2/20-4/15,3/13
Ugii Lake	S	4	23	3/27-4/29.4/14	4/5-5/19,5/4
Ulungur Lake	B	4	46	4/7-5/7,4/21	4/17-8/31,6/25
Buuntsagaan Lake	S	2	44	4/2-6/8	4/7-8/17
Khovsgol Lake	B	2	5	4/5-4/18	4/7-5/4
Dood Targan Lake	B	2	36	5/17-5/22	8/1-8/11
Khar Us Lake	B	2	18	3/25-5/9	/
Uvsiin Khar Us Lake	B	2	22	3/25-3/27,3/26	4/18
Terkhiin Tsagaan Lake	B	1	43	4/29	6/11
Erkhel Lake	S	1	42	5/16	7/12
Sangiin Dalai Lake	B	1	22	4/19	5/11
Telmen Nur	B	1	/	6/27	/
Shargiin Tsagaan Lake	B	1	37	4/22	5/29
Hyargas Nur	B	1	82	6/11	9/1
Olgoi Nur	B	1	30	5/10	6/9

In Mongolia, breeding or molting sites of whooper swans ranged from the westernmost Khovd River basin to the central Gobi wetlands of Ongiyn Gol, Baydrag Gol and the Tuin River, as well as northern-central basins of the Orkhon River, Tuul River and Selenga River, and the northernmost Shinshkhed River basin. Throughout these river basins, some lakes and rivers served as important sites with higher location densities or longer durations for swans during breeding or molting periods based on the tracking data (Table 1).

### Spatiotemporal analysis of BBMM and H5N1 outbreak

**Figure 2:**
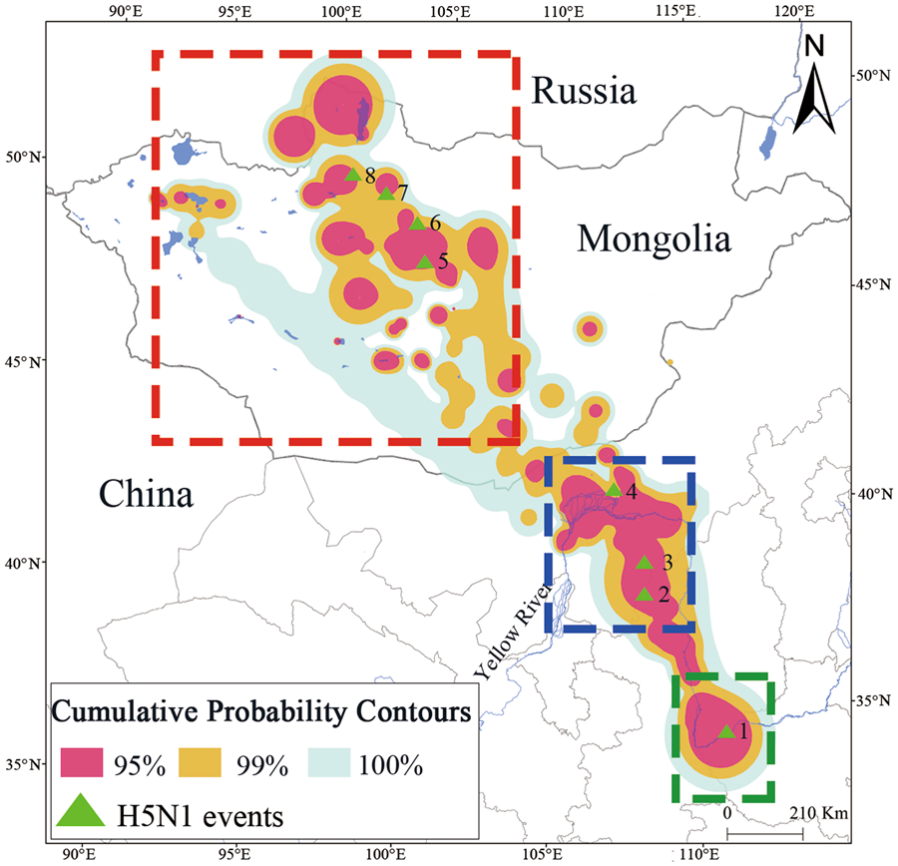
Brownian bridge movement model during the spring migration of whooper swans wintering at Sanmenxia Reservoir. H5N1 events: 1. Sanmenxia Reservoir area. 2. Hekou Reservoir. 3. Tsagaan Nur of Ordos. 4. Wuliangsu Lake. 5 Doitiin Tsagaan Lake. 6. Khunt Lake. 7. Doroo Nuur. 8. Erhel Lake. Different color boxes show the different months of whooper swan migration. Months have some overlap owing to individual differences in whooper swans migration. Dark blue boxes, dark green boxes and red boxes represent January to February, February to May and May to September, respectively.

BBMM shows that whooper swans wintering at the Sanmenxia Reservoir area occupied more space in China and Mongolia (Fig. 2) during the migration. At the sites that experienced H5N1 event outbreaks, in May 2015, several hundreds of wild fowl including wild swans and ducks that died were infected with H5N1 at Wuliangsu Lake and Tsagaan Nur, areas that sit exactly on the core use area of the 95% contour level for whooper swans and where there was a high population density distribution zone that was exactly identical with the migration data of whooper swans. Similarly, we also found there was a high degree of spatiotemporal overlap between the core use area of the 95% contour level for the spring migration pathway and historical H5N1 events including Doitiin tsagaan Lake, Khunt Lake, Saikhan Soum, and Erhel Lake in Mongolia, which sit on central-north river and lake wetlands, from 2005 to 2010 and exactly in the breeding period of whooper swans on the basis of tracking date.

We not only analyzed the relationship between the H5N1 events and home range but also evaluated the risk factors in epidemic diffusion. There was a significant difference in the distribution of the H5N1 events and random points in 95%, 99% and 100% of the home ranges (*P* < 0.01). It can be seen from Table 2 that there is a substantial relationship between the home range and the H5N1 events (OR > 1). The probability of outbreaks in 95%, 99% and 100% of the home range is respectively 57.00, 2.33 and 1.67 times greater than the probability of an outbreak outside the 95%, 99% and 100% of the home range.

**Table 2:** Relationship between presence of H5N1 cases and the home range of whooper swans at Sanmenxia Reservoir.

Home range	Univariate analysis	
	Crude OR (95% CI)	*P*-value
95%	57.00 (8.61–377.28)	<0.01
99%	2.33 (1.27–4.27)	<0.01
100%	1.67 (1.17–2.38)	<0.01

### Phylogenetics analysis

**Figure 3:**
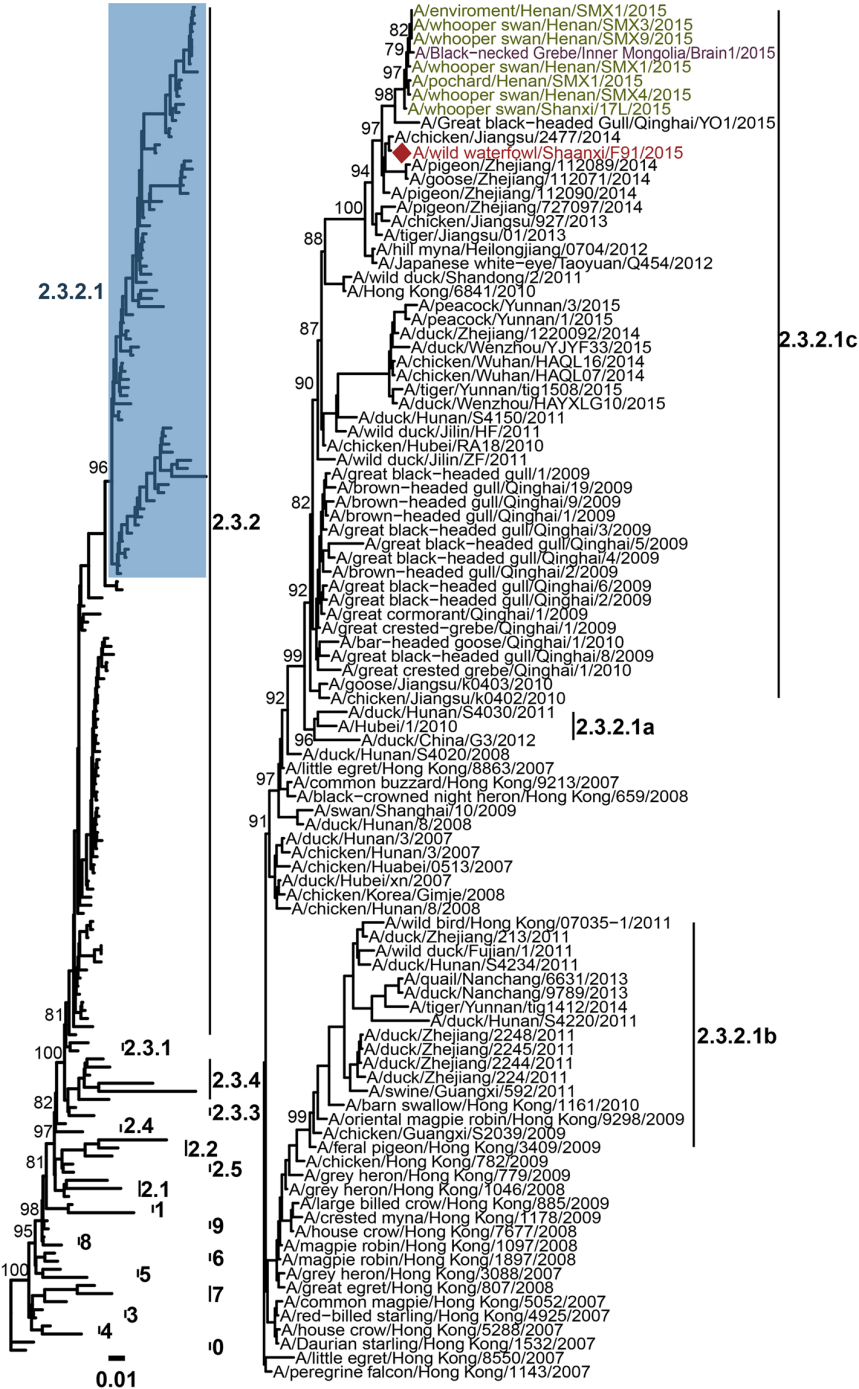
Maximum likelihood phylogenetic tree of HA sequences of HPAI H5N1 viruses and the subtree of clade 2.3.2.1. The virus isolated at Hekou Reservoir in Yulin of Shaanxi is indicated by a symbol (◆) and colored red. Viruses previously isolated at Sanmenxia Reservoir area were colored green. The virus isolated from a black-necked grebe in Inner Mongolia is colored purple.

Virology results for all 61 whooper swans were negative for AIV. Moreover, one influenza A (H5N1) virus, A/wild waterfowl/Shaanxi/F91/2015, was isolated from 3596 fresh fecal samples collected along the spring pathway of whooper swans at the Hekou Reservoir (Fig. 2) in April 2015. Phylogenetic analyses indicated that the HA segment of the strain belongs to clade 2.3.2.1c and has a high genetic homology with that of viruses previously isolated from whooper swans wintering in the Sanmenxia Reservoir area^21^ and a virus isolated from a black-necked grebe at Inner Mongolia (Fig. 3). This result suggests that whooper swans may transmit H5N1 viruses from the wintering site to stopover sites and possibly to breeding sites.

## Discussion

The population density of whooper swans at the Sanmenxia Swan Lake in the National Urban Wetland Park increased rapidly between 2010 (1.58 individuals/ha) and the winter of 2014 (32.11 individuals/ha). However, the subsequent decline to 13.92 individuals/ha during the wintering survey in December 2015 suggested that the population density may have been influenced by the AIV outbreak during the winter of 2014. The whooper swan was the dominant species at Swan Lake because of artificial provisioning with corn. The increased density of the whooper swans at the artificial feeding area greatly increased the chance of AIV spreading rapidly among individuals^2,3,22^. Therefore, it is believed that the relationship between population density and the size of feeding areas is important for H5N1 outbreaks. In addition, the total number of swans at the Sanmenxia Swan Lake in the National Urban Wetland Park in December 2015 was 24.3% lower than in 2014. This dynamic was similar to the population dynamics of the bar-headed geese *Anser indicus* that were infected by H5N1 in the 2005 breeding season, and the population declined significantly at Qinghai Lake in the 2006 breeding season^23^.

The intraspecific encounter probability increased with higher population densities, which carries a higher risk of spreading AIV, resulting in the outbreak of avian influenza, and subsequently, the encounter probability declined with a lower population density due to the outbreak of avian influenza, which may have been the result of population self-regulation. The swans had to balance their population dynamics through a “population increase–disease outbreak–population decrease” mechanism.

Our detailed summarization of the spring migration routes of whooper swans wintering in the middle reaches of the Yellow River in China and their summer breeding areas in central-northern and western Mongolia provides new baseline data for whooper swans in eastern Asia. Interestingly, we found migratory swans tended to use a narrower flyway during their earlier period of spring migration and subsequently dispersed when they arrived on breeding sites^16^. After departing the Sanmenxia Reservoir area, the majority of satellite-marked swans migrated intensively along the mainstem of the Yellow River and occupied more key stopover sites for up to 3 months. The minority of swans that flew across landscape barriers, such as the Kubuqi Desert, Maowusu Desert and Luliang Mountains, had fewer stopovers and a shorter migration time.

However, after a long period along the Yellow River in Inner Mongolia, swans expanded their migration course by flying to the central Orkhon River, Selenga River basin, and the northern Shinshkhed River basin as well as the western lakes along the Zavkhan River between the western Gobi Altai Mountains and Hangay Mountains, which cover a large area of Mongolia. We suspect that this funnel-like migratory trend may increase the risk of H5N1 dispersal.

Of the 61 tracked swans, 58 selected the upper reaches of the Yellow River in Inner Mongolia for their stopovers, where they foraged and rested for the longest period (24.74 ± 9.38 d). In March 2015 and April 2016, we observed that there was a large area of intensive farmland along the Yellow River that tens of thousands of swans occupied. In March and April every year, this farmland that consists of crops including sunflower and corn is full of water from the Yellow River and forms a flood plain that is favorable for whooper swans to rest and forage. Additionally, the wetlands are covered with reeds that provide good shelter for wild waterbirds and experience less human disturbance, which is likely why swans preferred to remain here for extended periods.

Takekawa *et al*. analyzed the spring migration routes of waterfowl wintering at Poyang Lake, China, using satellite tracking data^24^. Of the eight tagged birds, one falcated teal *Anas falcata* flew to the upper reaches of the Yellow River in Inner Mongolia instead of following the main migration routes crossing the Bohai Sea to Russia, which is one of the important stopovers of whooper swans. Therefore, it appears that some waterfowl wintering at the Yangtze River migrate to the upper reaches of the Yellow River and aggregate with whooper swans. The waterfowl using the Central Asia Flyway, such as bar-headed goose from Qinghai Lake, flew to breeding sites in central Mongolia by also crossing whooper swan migratory stopover locations such as Juyan Lake in western Inner Mongolia^25^. A total of 30,000 waterbirds, including whooper swans, whistling swans *Cygnus columbianus*, bar-headed geese, ruddy shelducks *Tadorna ferruginea*, and swan geese *Anser cygnoides*, were recorded during the survey of the upper reaches of the Yellow River from April 2015 to March 2016. This shows that the Yellow River in Inner Mongolia is the key convergence zone where waterbirds from eastern China and central Asia aggregate, indicating a high risk of H5N1 spread and evolution^26^.

It should be noted that the HA gene segment of the virus isolated from domestic ducks in Zhejiang and Jiangsu in eastern China was even more closely related to the virus isolated in this study from wild waterfowl in the upper reaches of the Yellow River. This indicates possible cross-species transmission. The whooper swans are widely distributed in eastern China, such as in the Zhejiang and Jiangsu Province^27^, where there exists considerable backyard and free-grazing poultry production. The infected waterfowl and whooper swans could have migrated to the Yulin and Ordos regions, the upper reaches of the Yellow River, in the spring and gathered with waterfowl migrating from the middle and west China, or even central Asia, resulting in virus transmission and evolution. The results provide important evidence regarding virus transmission along the migratory route, which connects the poultry culture zone in eastern China to the northern breeding ground.

Wuliangsuhai Lake and the Ordos Lakes in Inner Mongolia were identified as important stopovers for migrating whooper swans. In May 2015, wild ducks were infected with H5N1 at both of these locations. It is believed that the presence of the virus at the wetlands enabled infection to other waterfowl during the migration period^4,17^ and that the whooper swans increased the potential risk of spreading the virus during their migration^5^.

At whooper swan breeding sites in central Mongolia, H5N1 was isolated from individuals between 2005 and 2010. These breeding sites, such as Khunt Lake, Erkhel Lake, and Doityn Tsagaan Lake, are located at the breeding sites of the spring migration. Satellite data showed that the swans moved extensively around these lakes and rivers, which may have increased the risk of virus dispersal. The wetlands, which provide habitat for many waterfowl, are important factors that affect the spread of the virus. Therefore, the wetlands identified as key molting or breeding sites should be used as the main AIV surveillance sites.

Prosser *et al*. confirmed that bar-headed geese, ruddy shelducks and other waterfowl crossing Qinghai Lake migrate to breeding sites in central Mongolia along the Central Asia Flyway^25^. According to the tracking data in our study, the same breeding sites were also selected by whooper swans. Therefore, there is an increased risk of AIV transmission between Qinghai Lake in western China and Sanmenxia in central China^28^. A similar increased risk appears to exist for other regions.

Through the regression analysis of the H5N1 events and the home range of whooper swan, we can see that avian influenza outbreaks are closely related to bird activity. Wild birds might stop for water and food at some key stopover sites that include the upper reaches of the Yellow River, Bayannur and Ordos in Inner Mongolia, and Yulin in Shaanxi Province along their migration flyways, promoting virus transmission among birds^29,30^. Thus, the denser the bird activity is, the higher the risk of bird flu outbreaks. Studying the home range is very important to preventing the outbreak of avian influenza.

In this study, a strain of H5N1 2.3.2.1.c subtype AIV was isolated from Yulin in Shaanxi, which is key stopover site for whooper swans during their spring migration. The results of the phylogenetic analysis showed that the strain has a high degree of homology (99.7%) with the epidemic strain that we previously isolated from the Sanmenxia Reservoir area. We confirmed that whooper swans could carry the HPAI virus during migration, resulting in long-distance transmission.

In May 2015, at the period of spring migration for whooper swans, an H5N1 outbreak occurred at important stopovers of whooper swans in Inner Mongolia. Approximately one hundred black-necked grebes (*Podiceps nigricollis*) were found dead at Tsagaan Nur of Ordos^31^ (Fig. 2), and the H5N1 strain was closely related to sequences isolated from Sanmenxia, which further supports our conclusion.

## Methods

### Capture and marking

The Sanmenxia Reservoir area is located in the middle reaches of the Yellow River, central China (Fig. 1). In January and February from 2013 to 2016, a large cage with food such as corn was established in the Sanmenxia Swan Lake to attract whooper swans. During the 3 years of the study, a total of 61 swans were captured with the approval of the State Forestry Administration of China. All swans were marked with metal rings and neck collars (white “A/G+number” on blue, for example, A57), and the 61 healthiest swans received satellite transmitters attached to their back with a harness (Table 3). We recorded body weight, age and sex for each swan. Age was classified as adult or juvenile based on visual observation. All swans were released within 30 minutes near the capture sites after processing. The 61 marked swans were denoted by the letters S, from S1 to S61 (Table 3).

**Table 3:** Marking information of whooper swans wintering in the Sanmenxia Reservoir area, central China from 2015 to 2016. A: Adult, J: Juvenile, M: Male, F: Female; “/” means no leg ring.

Satellite ID	Leg Ring	Collar	Age (Sex)	Days Signaled (d)	Satellite ID	Leg Ring	Collar	Age (Sex)	Days Signaled (d)
S01	Q00-5059	E60	A(/)	106	S32	Q00-6319	A22	A(F)	105
S02	Q00-6360	A70	A(F)	108	S33	Q00-6318	A21	J(F)	41
S03	Q00-6352	A67	J(M)	138	S34	Q00-6320	A23	A(M)	175
S04	Q00-6353	A77	J(M)	89	S35	Q00-6321	A24	J(M)	Tracking
S05	Q00-6359	A76	J(F)	Tracking	S36	Q00-6322	A25	A(F)	134
S06	Q00-6355	A73	A(F)	130	S37	Q00-6341	A44	J(F)	107
S07	Q00-6356	A75	J(F)	160	S38	Q00-6342	A45	J(F)	67
S08	/	E63	J(M)	114	S39	Q00-6323	A26	A(F)	77
S09	Q00-6358	A74	J(F)	162	S40	Q00-6324	A27	J(M)	88
S10	Q00-6354	A78	J(/)	150	S41	/	A60	J(/)	116
S11	Q00-6343	A46	A(F)	41	S42	Q00-6325	A28	J(F)	Tracking
S12	Q00-6344	A47	J(M)	184	S43	Q00-6326	A29	A(F)	45
S13	Q00-6345	A48	J(M)	Tracking	S44	Q00-6327	A30	A(M)	42
S14	Q00-6346	A49	J(M)	152	S45	Q00-6328	A31	A(F)	Tracking
S15	/	A61	J(/)	Tracking	S46	Q00-6329	A32	J(F)	66
S16	Q00-6347	A50	A(F)	Tracking	S47	Q00-6330	A33	J(M)	36
S17	/	A57	J(/)	Tracking	S48	Q00-6332	A35	J(M)	170
S18	Q00-6348	A51	A(M)	49	S49	Q00-6333	A36	J(M)	Tracking
S19	Q00-6349	A52	A(F)	Tracking	S50	Q00-6334	A37	J(F)	64
S20	Q00-6304	A07	J(F)	Tracking	S51	Q00-6335	A38	A(M)	58
S21	/	A55	J(/)	142	S52	Q00-6336	A39	A(F)	43
S22	Q00-6306	A09	J(F)	Tracking	S53	Q00-6338	A41	A(F)	43
S23	Q00-6307	A10	J(F)	58	S54	Q00-6339	A42	J(M)	67
S24	Q00-6308	A11	A(M)	98	S55	Q00-5051	A62	J(F)	181
S25	Q00-6309	A12	J(M)	118	S56	Q00-5052	A63	J(F)	Tracking
S26	Q00-6312	A15	A(M)	67	S57	Q00-5054	A65	J(F)	Tracking
S27	Q00-6313	A16	A(F)	Tracking	S58	Q00-5055	A66	A(F)	110
S28	Q00-6311	A14	J(F)	46	S59	Q00-5056	A68	J(F)	107
S29	Q00-6314	A17	A(F)	75	S60	Q00-5057	A69	A(M)	92
S30	Q00-6315	A18	A(F)	47	S61	Q00-5058	A71	J(F)	Tracking
S31	Q00-6316	A19	J(F)	175					

### Satellite telemetry information

Of the 61 solar-powered GPS transmitters weighing 30 g between 2014 and 2016, 51 transmitters were from Hunan Global Messenger Technology Co. Ltd. (Xiangtan, China) and 10 were from Tianjin Blueoceanix Technology Co. Ltd. (Tianjin, China). All devices weighed 0.2%–0.3% of the wintering swans’ body mass (average 9.8 kg). Location data of the GPS transmitters were acquired from the China Mobile Communication System at 1 location in 2 h intervals using GSM cards and were reported as latitude, longitude, and location time. The accuracy of the GPS transmitters was categorized into five classes: A (±5 m), B (±10 m), C (±20 m), D (±50 m), and invalid data. We restricted accuracy to A, B and C for our analysis.

### History of H5N1 outbreak information

Detailed H5N1 outbreak data, including longitude and latitude, date, species, and virus subtypes, were obtained from the United Nations Food and Agriculture Organization (http://www.fao.org) and the World Organization for Animal Health (http://www.oie.int).

### Sample collection and phylogenetics

Tracheal and cloacal swab samples were taken from the 61 captured whooper swans for laboratory testing. Field work was also carried out in March and April 2015 to collect fecal samples along the routes of the satellite tracked swans. All samples were stored at −80 °C and inoculated into 10-day-old embrocated specific-pathogen free (SPF) chicken eggs for virus isolation at the National Avian Influenza Reference Laboratory of China.

Hemagglutinin (HA) gene sequences of HPAI H5N1 viruses were obtained from the GenBank hosted by the National Center for Biotechnology Information (NCBI) as of 10 November 2016. Sequences were aligned with MAFFT v7.127b and trimmed to a length of 1716 bp. An approximate maximum likelihood tree was constructed by FastTree v2.1.4^32^ as the setting of GTR + Gamma with SH-like local-bootstrap support values to estimate the reliability of each split in the tree. The tree was rooted at A/goose/Guangdong/1/96 and structured according to the unified nomenclature system and virus clades for the HPAI H5N1 viruses of the World Health Organization.

### Spatial analysis

A stopover site was defined as an area where whooper swans stayed for a 72-h period or longer. A breeding site, breeding or/and molting site, indicated the northern limit of the spring migration route where swans stayed for more than 3 months. We classified the arrival date at the breeding site as the end of the spring migration for the swans. Hence, we only selected the GPS locations before the end of the spring migration for analysis. We classified swans that stayed in China in May and stopped migrating north as summer residents. Because the GPS signal was not always continuous, we defined the first quarter of the total period as the departure time and the last quarter as the arrival time, with the remainder as the flight or stopover time if the GPS signal was not continuous for a period of <5 consecutive days. Because the Yellow River in inner Mongolia as an important stopover site and ecological corridor where most of the whooper swans stayed for the longest time, we defined it to be migration critical and divided migration routes into 2 stages: (1) from wintering grounds to the Yellow River stretch in inner Mongolia, and (2) from the Yellow River stretch in inner Mongolia to breeding sites in Mongolia.

We ran the Brownian bridge movement model using the ‘BBMM’ package in Program R (version 3.4, R Development Core Team 2016) to estimate the population level of the 61 whooper swans in our data with requested contour levels of 95% and 99%^33,34^. The BBMM provides an empirical estimate of a movement path using discrete location data obtained at relatively short time intervals, which is the probability of being in an area during the time of observation that is conditioned on starting and ending locations. We calculated all whooper swan output grids at the same spatial extent and at a 200 × 1000 km^2^ grid resolution. We plotted and analyzed migration routes, time, and days at stopovers using ArcGIS version 10.2 (ESRI, Redlands, California, USA).

We set up 80 random points in the study area, and the ratio of random points to H5N1 events is 10 to 1. We used spatial analysis techniques for H5N1 events and random points in 95%, 99% and 100% of the home range by logistic regression in SPSS 20.0.

### Ethics statement

Approval for swan capture was granted by the State Forestry Department of Henan Province (No. 13 Yu Forest Protection [2017]).
